# P336: Safety culture in health institution of Ivory Coast

**DOI:** 10.1186/2047-2994-2-S1-P336

**Published:** 2013-06-20

**Authors:** O Oyourou, MF Adéoti

**Affiliations:** 1 National Institute of Public Health, Côte d'Ivoire; 2 University Felix Houphouet Boigny, Abidjan, Côte d'Ivoire

## Introduction

Culture of safety care refers to the values and beliefs shared by a group security, which is a common behavior. Its importance stems from the fact that there is a link between the perceptions of professionals and safety issues or reporting the frequency of occurrence of certain adverse events.

## Objectives

Evaluate the perception and attitudes of health professionals on the safety of services in three health facilities in Côte d'Ivoire.

## Methods

It is a cross-over study conducted in 2009 in three health institutions that are the National Blood Transfusion Centre (CNTS), The Public Health Pharmacy (PSP) and the Institute of Cardiology of Abidjan (ICA ). It focused on ten topics considered important for safety culture of care. The study was conducted using a questionnaire sent to health professionals. The score for each dimension corresponds to the average percentage of positive responses for each dimension. A score ≤ 50% indicates a dimension and to improve a score ≥ 75%, a size developed.

## Results

Of the ten dimensions studied, only the dimension of teamwork in the service an average score of 79.5% for the three health institutions with 72.5 for the PSP, 83.5% for the CNTS and 82.5% for ICA. CNTS and the PSP have a management support for security with respective scores of 80% and 75%. This is not the case of ICA (49.7%). These three structures do not have enough staff to cope with the workload (PSP 32%, CNTS: 45%, ICA: 25, 4%) These structures are also characterized by the lack of freedom of expression (PSP 33%, CNTS: 47%, ICA 53.3%) and poor organization of services does not allow an improvement in the quality of care (PSP 46.7%, CNTS: 53.3%, ICA 48.3%).

## Conclusion

These institutions are characterized by a spirit of mutual respect and within teams. However, there is a lack of communication and freedom of expression. The harmonization of behavior of all health professionals is an essential process in the process of care for patients.

## Disclosure of interest

None declared

